# Early and late-onset cell migration from peripheral corneal endothelium

**DOI:** 10.1371/journal.pone.0285609

**Published:** 2023-05-10

**Authors:** Alina Miron, Sorcha Ní Dhubhghaill, Viridiana Kocaba, Martine J. Jager, Gerrit R. J. Melles, Silke Oellerich

**Affiliations:** 1 Netherlands Institute for Innovative Ocular Surgery, Rotterdam, The Netherlands; 2 Amnitrans EyeBank Rotterdam, Rotterdam, The Netherlands; 3 Department of Ophthalmology, Leiden University Medical Center, Leiden, The Netherlands; 4 Department of Ophthalmology, Antwerp University Hospital, Edegem, Belgium; 5 Melles Cornea Clinic Rotterdam, Rotterdam, The Netherlands; 6 Singapore Eye Research Institute, Tissue and Cell Therapy Group, Singapore, Singapore; Singapore Eye Research Institute, SINGAPORE

## Abstract

In this study we describe peripheral corneal endothelial cell migration *in vitro* in the absence and presence of a ROCK-inhibitor. For this study, 21 corneal endothelial graft rims, with attached trabecular meshwork (TM), were prepared from Descemet membrane-endothelial cell sheets by 6.5 mm trepanation. For the initial proof-of-concept, 7 outer graft rims were cultured in a thermo-reversible hydrogel matrix for up to 47 days. To assess the effect of a ROCK-inhibitor, 14 paired outer rims were cultured either with or without ROCK-inhibitor for up to 46 days. At the end of culture, tissue was retrieved from the hydrogel matrix and examined for cell viability and expression of different endothelial cell markers (ZO-1, Na^+^/K^+^-ATPase, NCAM, glypican, and vimentin). All cultured rims remained viable and displayed either single regions (n = 5/21) or collective areas (n = 16/21) of cell migration, regardless of the presence or absence of ROCK-inhibition. Migration started after 4±2 days and continued for at least 29 days. The presence of ROCK-inhibitor seemed to contribute to a more regular cell morphology of migrating cells. In addition, 7 outer rims demonstrated a phenotypically distinct late-onset but fast-growing cell population emerging from the area close to the limbus. These cells emerged after 3 weeks of culture and appeared less differentiated compared to other areas of migration. Immunostaining showed that migrated cells maintained the expression patterns of endothelial cell markers. In conclusion, we observed 2 morphologically distinct migrating cell populations with the first type being triggered by a broken physical barrier, which disrupted contact inhibition and the second, late-onset type showing a higher proliferative capacity though appearing less differentiated. This cell subpopulation appeared to be mediated by stimuli other than loss of contact inhibition and ROCK-inhibitor presence. Further exploration of the differences between these cell types may assist in optimizing regenerative treatment options for endothelial diseases.

## Introduction

The treatment of corneal endothelial dysfunction is transitioning from corneal transplantation towards regenerative therapy [1–3]. Since central corneal endothelial cells are considered terminally differentiated and non-replicative *in vivo* [4], new therapies that rely on the presence of healthy peripheral endothelial cells, such as Descemet stripping only (DSO), require endothelial migration from the periphery to cover the central area and endothelial cell mitosis through the administration of mitogens [5–9]. During this wound healing process, endothelial cells deposit fibronectin and laminin on the Descemet membrane which supports the required signaling for directed cell migration. Cells undergo cytoskeletal changes during the migration process reflected by cellular enlargement and polymorphism. Since the endothelial cells usually do not replicate *in vivo*, cellular enlargement is needed to cover the wounded areas. It has been suggested, however, that cells from the peripheral corneal endothelium may have proliferative capacity and act as a cell resource for the recovery of corneal endothelium in endothelial injury [10]. When immunostained, peripheral endothelial cells exhibit less differentiation markers than central endothelial cells but express stem cell markers and, sometimes, proliferation markers [11]. Furthermore, these cells were found between the corneal endothelium and the trabecular meshwork (TM) and showed a bipolar, spindle-shaped morphology similar to that of neural crest cells [12]. Recently, progenitor-like cells were discovered to reside within a thin strip of tissue, the transition zone (TZ), initially believed to be a zone depleted of cells [13,14]. These stem cells sequestered in specific niches inside the TZ may respond to corneal wounding to initiate endothelial repair and also contribute to a normal, slow replacement of corneal endothelial cells.

Endothelial cell migration can be supported by topical administration of a ROCK-inhibitor, which resulted in improved clinical outcomes after DSO [15–20]. The activation of the RhoA/Rho kinase (ROCK) pathway is associated to a multitude of cellular events ranging from actin cytoskeleton organization, cellular adhesion, cytokinesis modulation and it also affects regulation of proliferation and apoptosis. These effects play important roles in modulating the adhesion and migration of CECs during tissue repair and are fundamentally important for proliferation and protein production [5].

The purpose of this study is to evaluate *in vitro* peripheral endothelial cell migration from outer corneal graft rims using a thermo-reversible temperature-responsive polymer culture technique both in the absence and presence of a ROCK-inhibitor to gain a better understanding of the migration process *in vivo*.

## Materials and methods

### Corneas

Twenty-one human postmortem corneas, which were ineligible for transplantation, but had an intact and viable endothelial cell layer with an average endothelial cell density of 2371 (±313) cells/mm^2^ (from twelve donors (mean age 76 (±5) years; range 68–84 years)) (**S1 Fig and Table 1**), were obtained from Amnitrans EyeBank Rotterdam (www.amnitrans.nl, contact: info@niios.com). All donors had previously stated that they had no objection to transplant-related research. No specific institutional review board approval was required as currently the national law on medical-scientific research on human subjects does not apply to research with post-mortem human donor tissue if donors had not objected to having the samples used for research purposes (https://english.ccmo.nl/investigators/legal-framework-for-medical-scientific-research/your-research-is-it-subject-to-the-wmo-or-not).

**Table 1:** Demographics of donor corneas.

# Cornea (Donor)	Donor age (years)	Gender	Cause of death	Diabetic status	Sepsis status	Preserv. time before DM-EC sheet prep. (T_1_) (days)	Preserv. time between T_1_ and tissue embedding (days)	ECD (cells/mm^2^) at cornea preserv.	Rejection reason	DM-EC sheet Cell migration		
										4 mm circular graft	Outer rim graft	
**Proof-of-concept cell migration study**												
1 (1 R)	81	M	Circulatory syst.	No	No	14	6	2200	PEQ	CM	SCM	
2 (2 L)	80	M	Circulatory syst.	Yes	No	12	2	2700	Other	CM	SCM	
3 (3 R)	74	M	Circulatory syst.	No	No	16	7	2500	PEQ	n.v.	CM	
4 (4 R)	81	F	Circulatory syst.	No	No	1	2	2500	Virology	n.v.	CM	
5 (4 L)	81	F	Circulatory syst.	No	No	1	2	2500	Virology	CM	CM	
6 (5R)	71	M	Circulatory syst.	No	Yes	12	2	2600	Screening tests	n.v.	CM	
7 (5L)	71	M	Circulatory syst.	No	Yes	12	2	2600	Screening tests	CM	CM	
**Mean (±SD)**	**77 (±5)**					**11 (±6)**	**4 (±2)**	**2514 (±157)**				
**Paired outer graft rims cell migration study**												
									**ROCK inhibitor**	**Cell migration**	**Late-onset cells**	
8 (6 R)	84	M	Respiratory sys.	No	Yes	21	2	2300	Other	No	SCM	No
9 (6 L)	84	M	Respiratory sys.	No	Yes	21	2	2300	Other	Yes	CM	No
10 (7 R)	80	M	Respiratory sys.	Yes	No	22	2	2400	PEQ	Yes	CM	Yes
11 (7 L)	80	M	Respiratory sys.	Yes	No	22	2	2400	PEQ	No	CM	No
12 (8 R)	77	M	Other	Yes	Yes	24	2	2200	Virology	No	CM	Yes
13 (8 L)	77	M	Other	Yes	Yes	24	2	2100	Virology	Yes	SCM	No
14 (9 R)	78	M	Malign.	No	No	4	4	1800	PEQ	No	CM	Yes
15 (9 L)	78	M	Malign	No	No	4	4	1800	PEQ	Yes	CM	Yes
16 (10 R)	68	M	Circulatory syst.	Yes	No	10	3	3000	Virology	Yes	CM	No
17 (10 L)	68	M	Circulatory syst.	Yes	No	10	3	2900	Virology	No	CM	No
18 (11 R)	68	M	Digestive syst.	No	No	15	2	2400	Virology	Yes	CM	Yes
19 (11 L)	68	M	Digestive syst.	No	No	15	2	2400	Virology	No	CM	No
20 (12 R)	75	M	Malign.	No	No	24	2	2300	PEQ	No	CM	Yes
21 (12 L)	75	M	Malign	No	No	24	2	1900	PEQ	Yes	CM	Yes
**Mean (±SD)**	**76 (±6)**					**17 (±8)**	**2 (±1)**	**2320 (±347)**				
**Mean all grafts (±SD)**	**76 (±5)**					**13 (±7)**	**3(±2)**	**2371 (±313)**				

CM: Collective migration

DM: Descemet membrane

EC: Endothelial cells

ECD: Endothelial cell density

F: Female

L: Left donor cornea

M: Male

Malign.: Malignant

n.v.: Not viable

PEQ: Poor Endothelial Quality

Prep.: Preparation

Preserv.: Preservation

R: Right donor cornea

SCM: Single cell migration

Screening test: Inconclusive blood test

SD: Standard deviation

syst.: System

Virology: Hepatitis B positive.

### Outer graft rim preparation

Corneoscleral buttons were excised from whole donor globes obtained less than 24 hours postmortem and stored in organ culture medium at 31°C (CorneaMax, Eurobio, Courtaboeuf, France) until graft preparation. The Descemet membrane-endothelial cell (DM-EC) sheets were separated from the stroma by using the standardized “no-touch” peeling technique, as described previously [21,22]. After placing the isolated DM-EC sheets with the trabecular meshwork (TM) still attached on a soft contact lens, the DM-EC sheets were centrally trephined with a 4 mm biopsy punch followed by a second concentric 6.5 mm trepanation to obtain the outer rims. Attachment of the TM prevented the outer rims from scrolling and facilitated further handling. Outer graft rims and 4 mm circular DM-EC sheets were stored separately in growth factor-depleted DMEM-based medium (M1) (**Table 2**) for 2 to 7 days before culture in the thermo-reversible hydrogel matrix (Mebiol Gel, Cosmo Bio, USA).

**Table 2:** Composition of the culture media used in this study.

Culture medium	Growth factors and supplements
Growth factor-depleted DMEM-based medium (M1)	• DMEM • 15% fetal bovine serum (FBS) • 2 mM L-Glutamine (L-Glu)
DMEM based culture medium (M2):	• 15% fetal bovine serum (FBS) • 2 mM L-Glutamine (L-Glu) • 2 ng/ml basic fibroblast growth factor (bFGF) • 0.3 mM L-Ascorbic acid 2-phosphate (Asc-2P) • 10,000 U-ml Penicillin-Streptomycin (Pen/Strep)
ROCK-inhibitor enriched medium (M3):	• M2 + 10 μM ROCK-inhibitor

FBS and bFGF were purchased from ThermoFisher Scientific Europe BV

The Netherlands; DMEM, ROCK-inhibitor, L-Glu and Asc-2P were obtained from Sigma-Aldrich Chemie NV, The Netherlands; Pen/Strep was bought from Carl Roth GmbH, Karlsruhe, Germany.

**Fig 1:**
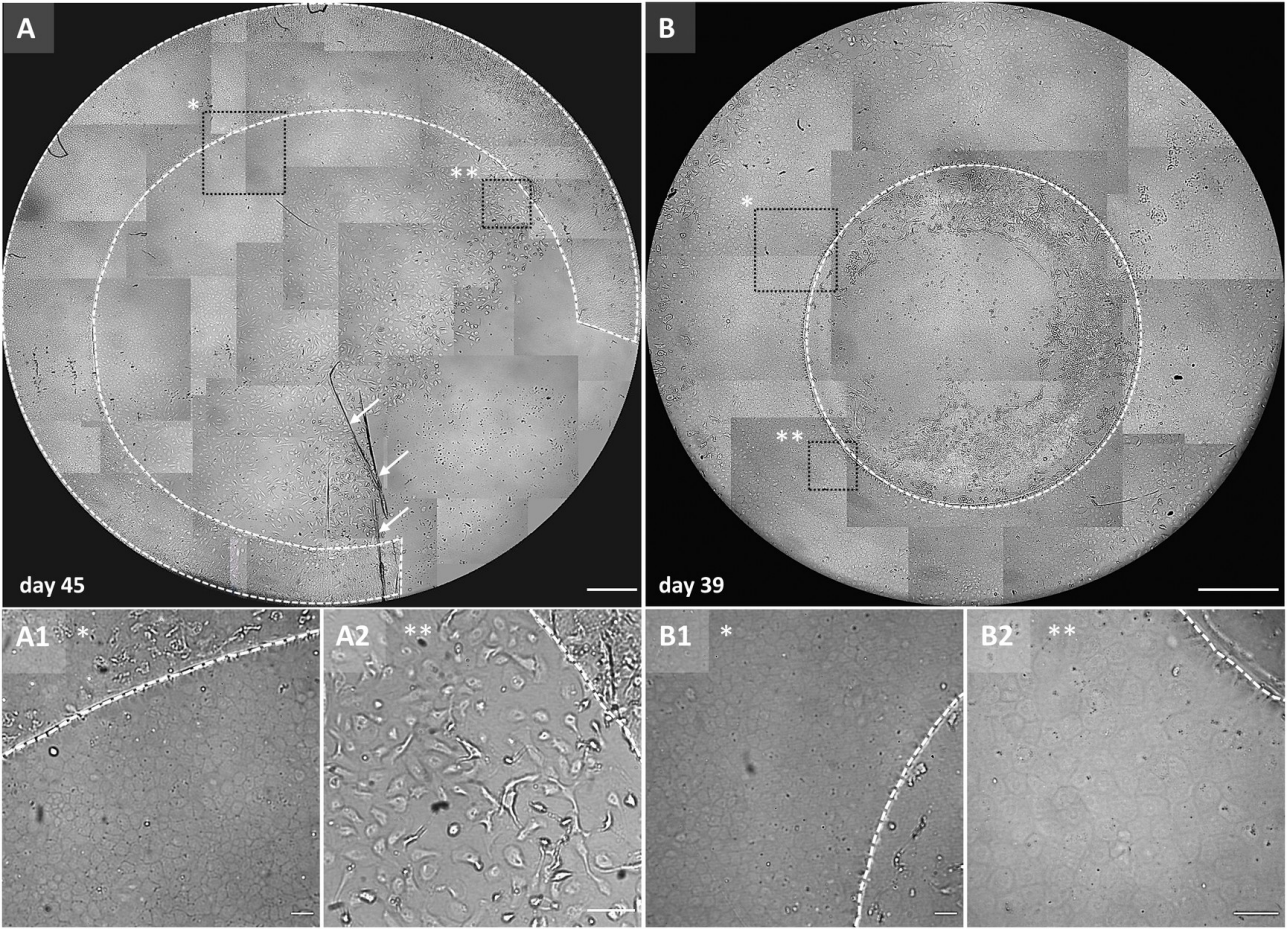
Outer rim and a central graft representation of the proof-of-concept migration study. Collage of light microscopy images (x50) to create an overview of the (A) outer rim cell migration (Cornea #7, Table 1) after 45 days in gel culture for which the ring opening was performed in the well using a surgical blade (note the imprints highlighted by the white arrows). The graft ring showed predominantly collective cell migration resulting in the formations of a cell monolayer free from the DM substrate as depicted in the squared area marked with an asterisk in the overview image and enlarged in (A1), while the higher magnification image (A2) marked by ** in the overview image, shows some migration of individual cells to illustrate the heterogeneity of migration from the graft rims. (B) 4 mm control graft after 39 days in gel culture showing uniform cell migration from all around the round edges; two higher magnification images marked by * (B1) and ** (B2) in the graft overview show the homogeneous cell monolayer formed by the collective spreading of cell cohorts. The dotted line outlines the outer rim (A), control graft (B), and cell monolayer migration edge in the higher magnified images. The scale bars in (A) and (B) is 1000 μm, and in (A1), (A2), (B1), and (B2) is 100 μm.

A thermo-reversible cell culture system not only allows for studying endothelial cell migration but also facilitates tissue retrieval, without enzymatic treatment, by cooling the gel below the sol-gel transition temperature [23]. The preparation of the thermo-reversible hydrogel matrix and embedding protocol have been described in previous publications from our group [23,24]. In brief, a thin layer of liquified gel is slowly added over the center of the explant tissue. Once firm, more liquified gel was added up to a volume of approximately 250 μl for a 48-well plate, and 500 μl for a 24-well plate. Subsequent incubation at 37°C for about 5 minutes led to a solidified gel matrix uniformly distributed over the explants. Growth factors and nutrients were replenished every 2–3 days by keeping the gel surface moist with 150 μl of culture medium for a 48-well plate and 300 μl for a 24-well plate. When embedding the tissue in the hydrogel matrix, the 4 mm circular DM-EC sheets were placed, endothelial-side-up, on glass coverslips and transferred to a 48-well plate (well diameter 11.0 mm) [25]. The outer graft rims were transferred to a 24-well plate (well diameter 15.6 mm) containing 100 μl Dulbecco’s phosphate-buffered saline (PBS, Sigma-Aldrich Chemie NV, The Netherlands) and positioned endothelial-side-up by grasping the TM with a forceps. The surface was then carefully dried out with cellulose vitreous sponges and the graft rim was carefully cut radially with a surgical blade (Swann-Morton, Sheffield, England) to ensure that the graft rims were mounted completely flat on the surface. Hypotonic Trypan Blue solution 0.04% (Hippocratech, Rotterdam, The Netherlands) was used to ensure the visibility of both the rims and circular grafts during preparation and unfolding on the solid support. For the initial proof-of-concept experiments, 7 outer graft rims and the corresponding 4 mm circular grafts were embedded separately inside the thermo-reversible polymer solution and evaluated for chemotactic cell ability (**Fig 1**). The central excised circular grafts acted as the control for cell migration [23]. Following the proof-of-concept, 7 paired outer rims were cultured in the presence or absence of ROCK-inhibitor to assess the effect of ROCK-inhibitor (Y-27632, Sigma-Aldrich Chemie NV, The Netherlands) on the *in vitro* cell migration. The tissue was cultured up to 47 days (proof-of-concept) and 46 days (paired study), respectively, in a humidified atmosphere at 37°C with 5% CO_2_. Cultures were observed every 2–3 days when the DMEM based culture medium (M2) and ROCK-inhibitor enriched medium (M3) (**Table 2**) were refreshed. Corneal endothelial cell morphology and migration were examined with an AxioVert.A1 microscope with AxioCam 305 color camera (Zeiss, Oberkochen, Germany). Cell migration was either labelled as collective cell migration or as single cell migration. Collective cell migration was defined as collective motion of a cohesive cell group that remains connected throughout the migration process, while single cell migration is characterized by a lack of cell-cell junction of the migrating cells. Cases displaying both collective migration and single cell migration (**Fig 1**), were labelled as ‘collective migration’ (CM) in **Table 1**. The recovery of the tissue from the hydrogel matrix at the end of the culture period was performed by gradually cooling the gel below the sol-gel transition temperature (<20°C) using low-temperature PBS in the manner previously described [23].

### Immunohistochemistry and cell viability analysis

After the removal of the hydrogel, samples were subjected either to immunohistochemistry analysis with several markers such as tight junction protein zonula occludens (ZO-1), vimentin, neural cell adhesion molecule (NCAM), glypican-4 (GPC4), and sodium-potassium pump (Na^+^/K^+^-ATPase) for routine cell characterization, or to cell viability analysis with Calcein-AM. As described in detail [24,25], tissue was first fixated in 4% paraformaldehyde (Sigma Aldrich, The Netherlands) for 15 minutes and then washed with PBS, permeabilized using a permeabilization buffer (0.1% Triton X-100 in PBS, Sigma Aldrich, The Netherlands) and finally incubated with blocking buffer (5% bovine serum albumin in PBS, Sigma Aldrich, The Netherlands) for 30 minutes in order to prevent non-specific staining. Incubation for 1 hour with primary antibodies (anti-ZO-1 tight junction protein (anti-ZO-1/TJP1, dilution 1:100; ThermoFisher Scientific Europe BV (Bleiswijk, The Netherlands)), anti-vimentin filamentous protein (anti-vimentin, dilution 1:100; Abcam (Cambridge, United Kingdom)), anti-neural cell adhesion molecule 1 protein (anti-NCAM, dilution 1:100; R&D Systems (Abingdon,United Kingdom)), anti-glypican 4 protein (anti-GPC4, dilution 1:100; Abcam (Cambridge, United Kingdom)), and anti-sodium/potassium-ATPase (anti-Na^+^/K^+^-ATPase β1, dilution 1:100; Sigma-Aldrich Chemie NV (Zwijndrecht, The Netherlands)) was followed by several PBS washing steps. Samples were then incubated with fluorescent secondary antibodies that had been conjugated to Alexa Fluor® (dilution 1:200; ThermoFisher Scientific Europe BV) for 45 minutes. Nuclear cell staining was performed with DNA-specific blue-fluorescent dyes DAPI (Sigma-Aldrich Chemie NV, The Netherlands) and Hoechst (ThermoFisher Scientific Europe BV, The Netherlands).

Calcein-AM (Sigma-Aldrich Chemie NV, The Netherlands) staining was performed to verify cell viability after hydrogel removal. Tissue was covered with 100–150 μl of PBS 4 μM Calcein-AM and incubated at room temperature for 45 min. After incubation, samples were washed with PBS and then imaged using an inverted fluorescence microscope (Axiovert.A1, Zeiss, Oberkochem, Germany).

## Results

### Corneal endothelial cell migration

#### Proof-of-concept extended culture study

Hydrogel embedding was successful for all 7 outer graft rims and allowed for observation of *in vitro* cell migration from day 2 up to day 47 during which cells migrated either only individually (2/7) or collectively (5/7) (**Fig 1**). For the two graft rings that showed only migration of individual cells, the corresponding 4 mm circular grafts showed collective cell migration (**S2 Fig**). Overall, graft rings showed some degree of heterogeneity in the coverage by an intact endothelial monolayer and especially for graft rings that displayed only single cell migration, the endothelial monolayer appeared compromised with some areas almost depleted of cells (**S2 Fig**).

#### ROCK-inhibitor effect on in vitro cell migration

**Fig 2:**
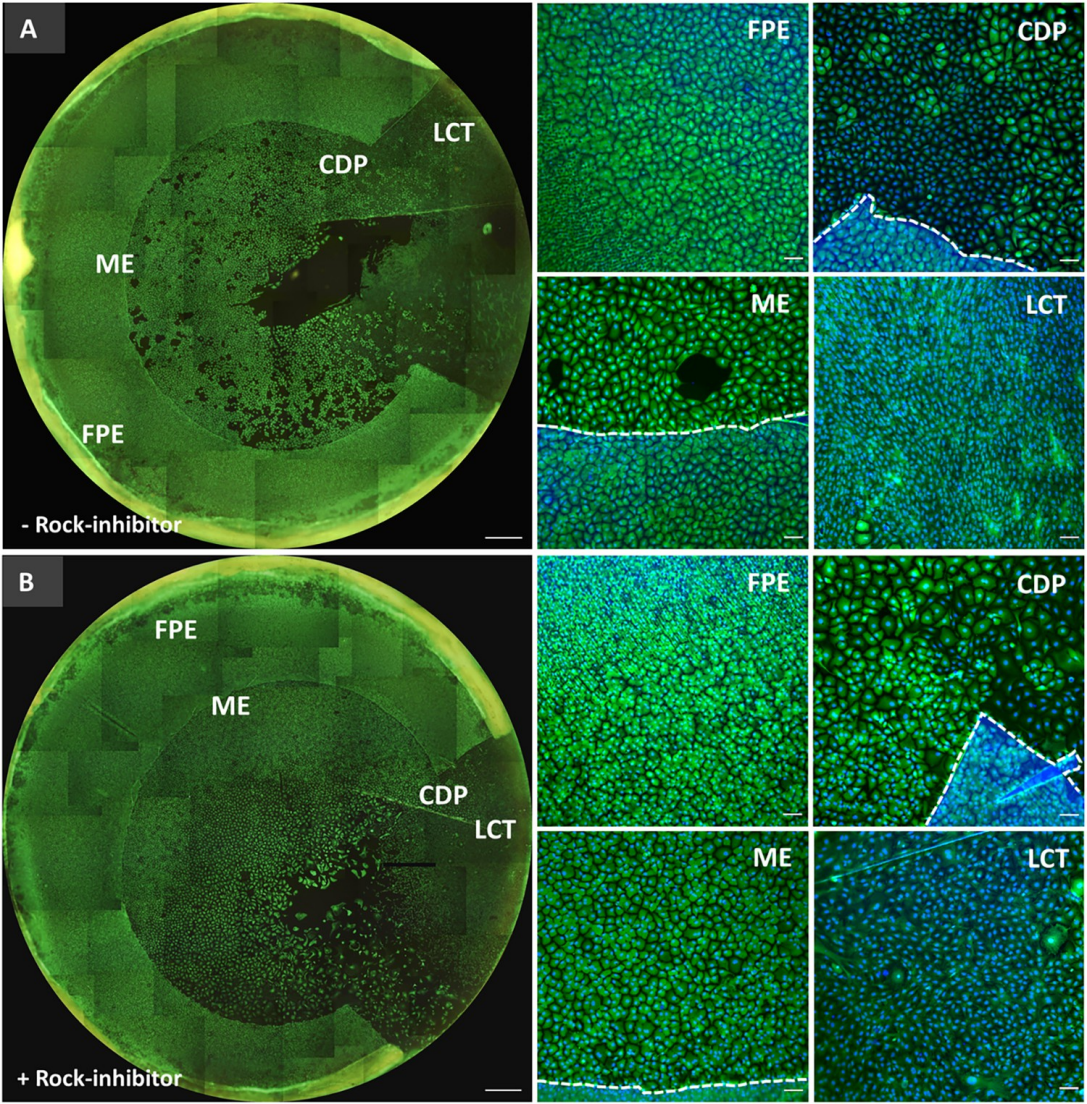
Calcein-AM and Hoechst staining of representative images of paired outer graft rims cultured in the (A) absence or (B) presence of ROCK-inhibitor. Displayed per culture condition are Calcein-AM and Hoechst fluorescence overlay images taken at culture day 45 of the far periphery endothelium (FPE), the cell monolayer migration edge (ME), cells of different phenotype (CDP) growing around the outer rim opening, and the late-onset cell type (LCT) with high proliferative capacity. The dashed lines outline the cell monolayer migration edges. Scale bars in (A) and (B): 1000 μm, and in higher magnification images: 100 μm.

Of seven outer graft rim pairs used to test the effect of the ROCK-inhibitor and cultured for up to 46 days, 5 pairs (**Table 1**) formed a continuous functional monolayer, as shown by the Calcein-AM and Hoechst staining (**Fig 2**). Mixed cell migration behaviour was observed for the other two pairs (**Table 1**). While one graft rim showed collective migration, the contralateral graft rim only displayed single cell migration. For one outer graft rim pair with collective migration initially, cells displayed a coordinated movement that turned into individual cell migration after approximately 17 days of culture. Moreover, cell migration was initiated from edges displaying a low density of viable cells, whereas it was absent from edges displaying higher densities of viable cells (**S3 Fig**). A similar migration pattern initiated from mechanically damaged areas was also observed for one outer graft rim cultured in the absence of ROCK-inhibitor (**S3 Fig**) whereas the contralateral graft rim showed a more uniform distributed migration along the open edges.

Overall, for pairs with collective migration in both graft rims, there was no remarkable difference in terms of the moment when the cell migration started (4±2 days), or the duration to maintain the direction of motion (44±2 days). The presence of ROCK-inhibitor in the culture medium seemed to contribute to a more regular cell morphology of the migrating cells and a migrating cell monolayer without significant formation of any gaps between cells (**Fig 2A, ME vs 2B, ME**).

**Fig 3:**
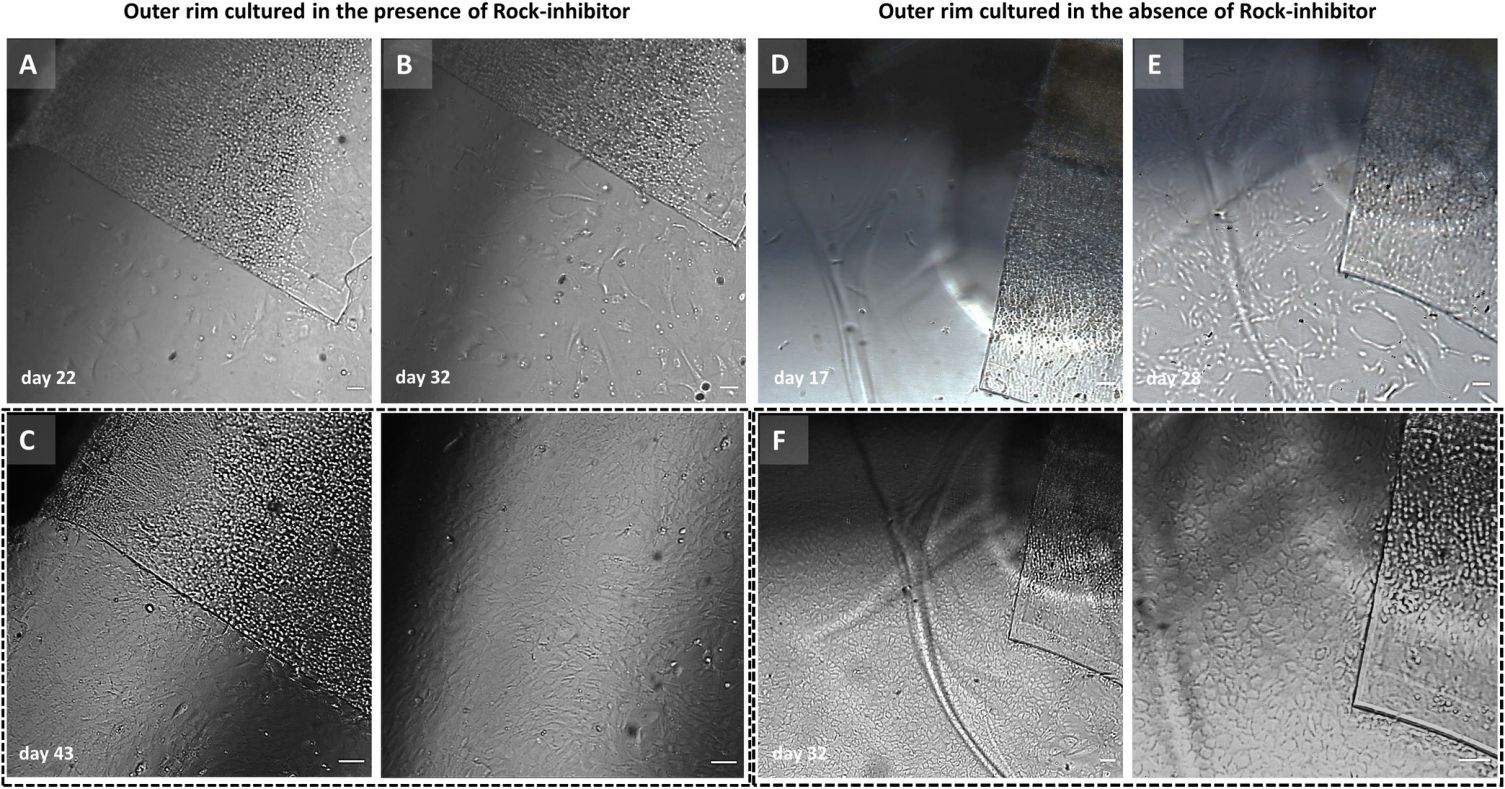
Representative images of two unpaired outer rims that displayed the late-onset cell type with high proliferative capacity. (A-C) Outer rim cultured in the presence of ROCK-inhibitor and the fast-growing cell type initiated after 32 days of culture but evident only at day 43. (D-F) Outer rim cultured without ROCK-inhibitor and the fast-growing cell type already visible after 28 days of gel culture. Scale bar: 100 μm.

**Fig 4:**
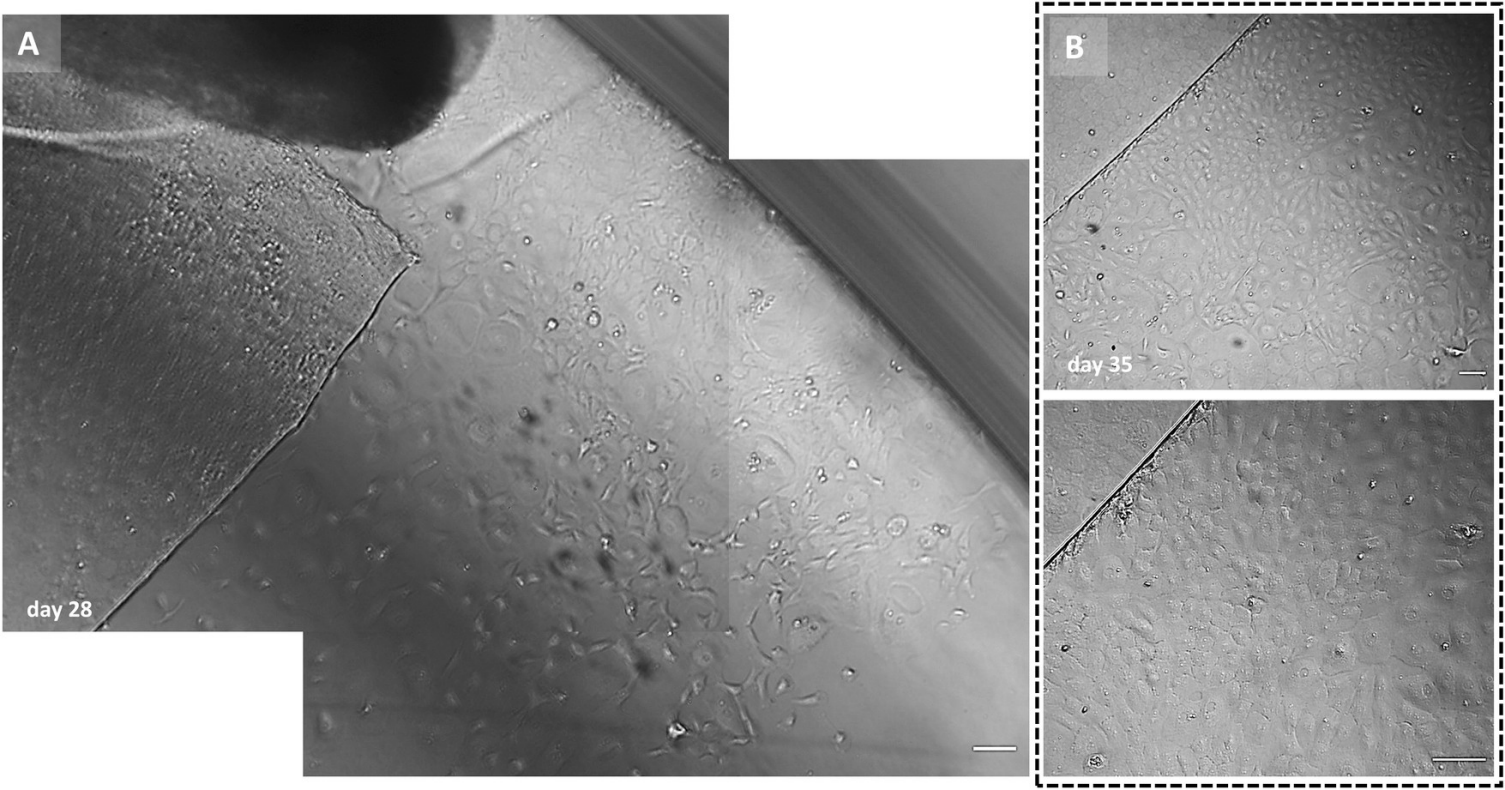
Example images of an outer rim displaying the late-onset cell population without actively targeting the Rho-kinase pathway. (A) Cell outgrowth in the far periphery of the endothelium visible after 28 days of gel culture. Note, (i) the different gel embedding plane of the TM that was not physically connected to the endothelium and (ii) the absence of cell migration from the radial cut edge of the outer rim. (B) After 35 days of gel culture, a new cell monolayer of optimal morphology formed around the rim’s cut edge. Scale bar: 100 μm.

Another finding, however, was that seven outer graft rims displayed a phenotypically distinct late-onset (but fast-growing) cell population emerging from the far periphery of the endothelium (**Fig 3**). The late onset migration was seen with both graft rings of 2 pairs, as well as with 3 unpaired graft rings (2 grafts in the presence and 1 in the absence of ROCK-inhibitor), while 2 other pairs did not show the late-onset cells at all. These late-onset cells started to migrate after 3–5 weeks of culture. Presence of ROCK-inhibitor in the growth medium did neither result in a higher percentage of graft rims displaying the late-onset cell population nor did it decrease the time-point when the migration capacity was unlocked (29±8 days with ROCK-inhibitor (n = 4) vs 27±3 days without ROCK-inhibitor (n = 3)). The cells emerged from the intermingled fibrillary area and acquired a more endothelial-like morphology when cultured in the absence of ROCK-inhibitor (**Figs 3D–3F and 4A**). These cells became contact inhibited and formed a monolayer of hexagonal cells within 10±4 days (in 4 of 7 cases) and 7±4 days (in 3 of 7 cases) of gel culture in the presence or absence of ROCK-inhibitor, respectively (**Figs 3C, 3F and 4B**).

#### Immunohistochemistry

**Fig 5:**
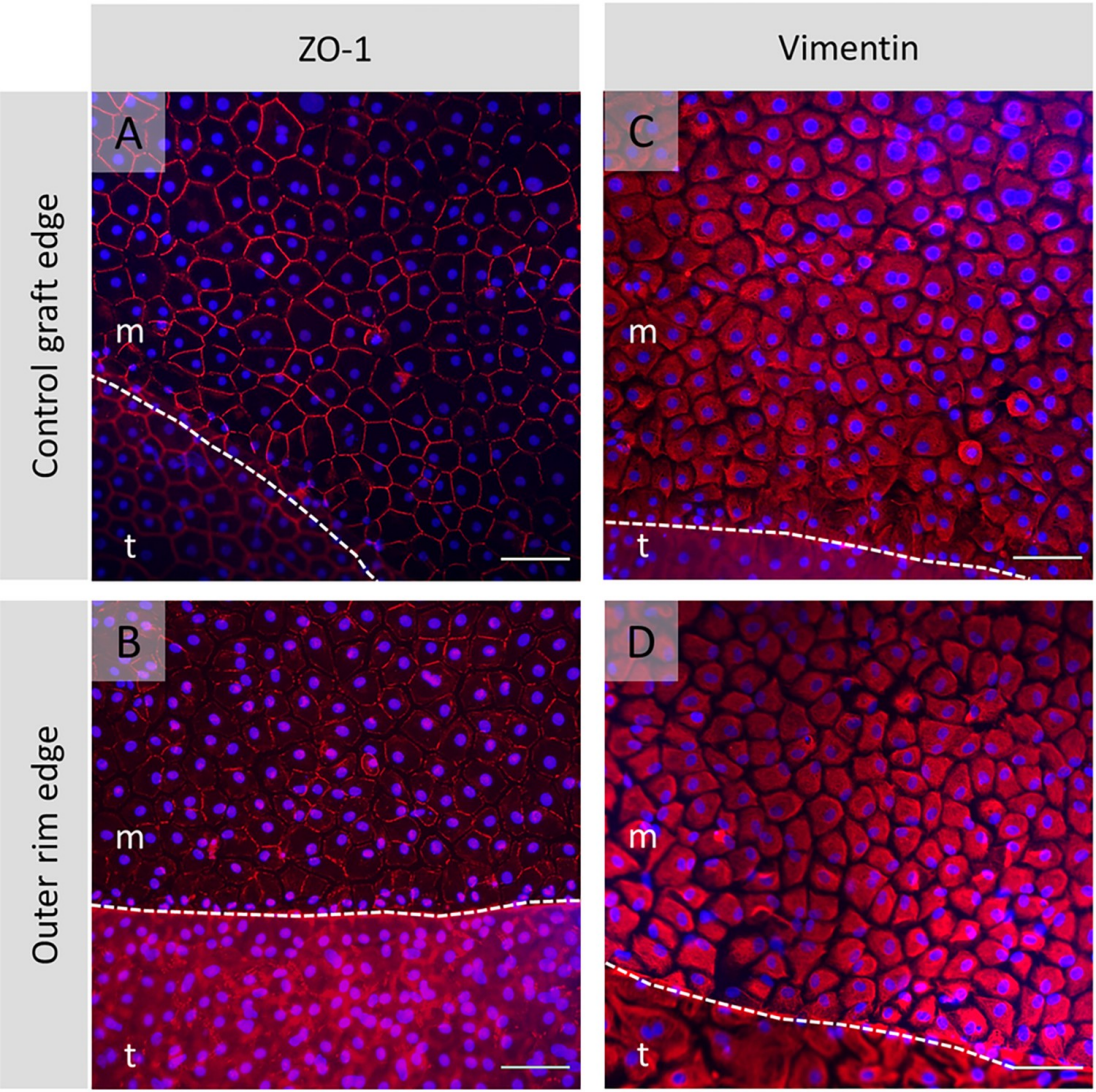
Representative images of structural proteins detected in the cell monolayer with focus on migration edge. Fluorescence microscopy images showing expression of ZO-1 (A, B: Red signal) and Vimentin (C, D: Red signal) counterstained with DAPI (blue signal) in the confluent layer of migrating cells: Control tissue (4 mm circular graft) (A and C) vs. outer graft rim (B and D). The dashed line outlines the cell monolayer migration edge that delineates the migrated cell monolayer (m) plane from the tissue (t) surface. Scale bar: 100 μm.

**Fig 6:**
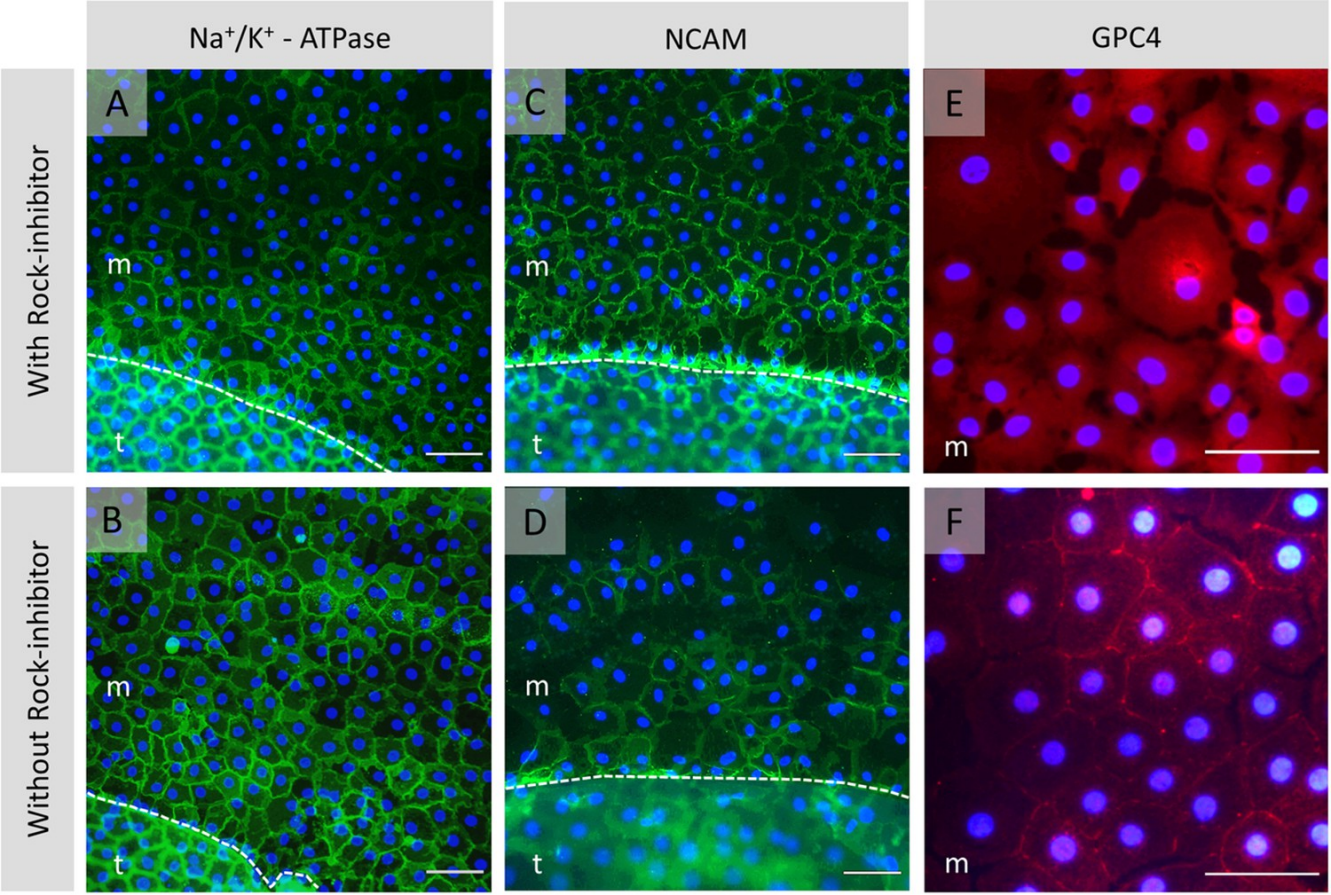
Immunofluorescence staining of the migrated monolayer from unpaired control grafts. Grafts were cultured with (top line: A, C, E) or without (bottom line: B, D, F) ROCK-inhibitor. Considerable expression of the functional cell marker Na^+^/K^+^-ATPase (A, B: Green signal), neural cell adhesion molecule NCAM (C, D: Green signal), and cell surface proteoglycan GPC4 (E, F: Green signal) was detected in all cultured monolayers irrespective of the culture media composition. Nuclei were stained blue by DAPI. The dashed line outlines the cell monolayer migration edge that delineates the migrated cell monolayer (m) plane from the tissue (t) surface. Scale bar: 100 μm.

After gel removal, immunohistochemistry analysis showed the typical expression profiles for the endothelial cell markers ZO-1 (**Fig 5A and 5B**) and Vimentin (**Fig 5C and 5D**) in the migrated cell monolayer. A weak signal was recorded for the functional marker Na^+^/K^+^-ATPase, adhesion marker NCAM, and cell surface marker GPC4 in the cell monolayer formed from the outer graft rim’s edge and tissue itself regardless of the culture medium composition (**Table 2. M2, M3**), while the 4 mm circular grafts revealed the formation of a continuous functional monolayer with tightly interconnected cells (**Fig 6**). No visual differences in the immunofluorescent expression patterns were detected between cultured monolayers formed by cell outgrowth from the 4 mm circular grafts with or without ROCK inhibition. Interestingly, cell viability evaluation of the seven outer graft rims with the late-onset cell population showed a heterogenous Calcein-AM signal intensity wherein the lowest signal detection corresponded to the cell population emerged from the far periphery of the endothelium (**Fig 2A: ME** vs **LCT** and 2**B: ME** vs **LCT**).

## Discussion

Corneal endothelial explant culture is technically challenging due to the inherent scrolling properties of the tissue, however, using a thermo-reversible hydrogel it is possible to selectively study *in vitro* corneal endothelial cell (CEC) migration from the outer regions of the monolayer with a central opening. Using this approach, we showed that CECs migrate collectively in the majority of outer graft rims with an intact endothelial monolayer. We observed early- and late-onset migration of two morphologically distinct CEC populations. The late-onset type relatively quickly covered a surface area larger than the tissue area it originated from without depleting the tissue of cells as shown by viability staining. Cell viability evaluation of outer graft rims with a late-onset cell population also showed a heterogenous Calcein-AM signal intensity wherein the late-onset cells displayed a lower fluorescence signal intensity than the cells that had started earlier with the migration. This may indicate that the late-onset cell population has a low intracellular esterase activity which can be interpreted as a reliable indicator of undifferentiated cells [26]. These findings suggest that the late-onset cell type displays a higher proliferative capacity though it appeared to be less differentiated. This cell subpopulation appeared to be mediated by stimuli other than the loss of contact inhibition and ROCK-inhibitor.

With both early- and late- onset migration continuous cell monolayers were formed with contact-inhibited cells and immunohistochemistry demonstrated the presence of viable cells with tightly packed morphologies. In some other areas, we also noted single cell migration with individual satellite cells branching into the free space, though these were most often seen from areas with mechanical damage. We suspect that the lack of collective migration from the neighboring undamaged endothelial areas may be partly due to inconsistency in flattening the tissue fixed on the support, trapping some regions in the gel during the solidified matrix formation.

Cultures of seven outer rims showed migration of a morphologically distinct, late-onset cell type which appears after 3–5 weeks in culture. These cells arose from the intermingled fibrillary area between the peripheral endothelium and TM. The late-onset cells first adopted a quickly migrating, fibroblast-like morphology (**Fig 3B and 3E**). Within 10 days of culture, however, the cell migration pattern became more coordinated in which cells acquired a CEC phenotype with a regular morphology (**Figs 3C, 3F** and **4B**). Interestingly, cell viability evaluation of outer graft rims with a late-onset cell population showed a heterogenous Calcein-AM signal intensity (**Fig 2A: ME vs LCT** and **2B: ME vs LCT**) where late-onset cells displayed a lower fluorescence signal intensity than the cells that had started earlier with the migration.

The late-onset cells appear to originate from the far peripheral area of the endothelium, a region that has been referred to as a progenitor enriched region (TZ) with the potential to generate mature human corneal EC [27,28]. For the far peripheral endothelium a high mitogenic activity has been reported due to their propensity to sphere formation when cultured on low adhesion surfaces [29]. When spheres were injected into the anterior chamber of rabbit eyes with corneal deficiency, they formed a functional endothelium [30,31]. Zhang et al. demonstrated that TZ cells can proliferate and differentiate into CECs by culturing TZ cells from donor corneal rims [32]. The TZ cells grew from the explant after 20 days of culture, exhibiting initially a rounder polygonal morphology, that become gradually more elongated and fibroblastic during passaging and finally polygonal 2 to 3 passages before senescence. As a general observation, terminal differentiation was identified when the TZ cells acquired round and polygonal morphology. While we cannot definitively state that the late-onset growth we observed represents TZ cell growth, the timing and end morphology of the cells is highly suggestive.

In our study, the observation of this late-onset cell population was only possible because the outer graft rims had been cut in order to flatten them on the substrate, however, the question is how these cells could be activated *in vivo*. While He et al. hypothesized that cells from the far periphery may have the ability to migrate towards the corneal center along DM grooves [11], it is yet still unknown whether the TZ/late-onset cells play an active functional role or can be stimulated to be functionally active *in vivo*.

Although endothelial-to-mesenchymal transition (EMT) has been described to occur when CECs are cultured over multiple passages and cells adopted a more elongated morphology [33], highly proliferative TZ cells first adopt a fibroblast-like morphology and later transform to a polygonal morphology [32]. Previous studies also reported that there is no association of the TZ dimension or proliferative characteristics with donor age, ethnicity, or cell density which is in agreement with our findings that the outer rims showing the late-onset cells were isolated from old donors (68–80-years-old) with variable ECD (1800–2400 cell/mm^2^), and preservation time before culture (8–26 days) [27].

Interestingly, the presence of ROCK-inhibitor in the culture medium did not alter the cell outgrowth from the outer graft rims. While it did appear beneficial for maintaining the cell shape and cell-cell adhesion contacts during collective migration, no differences in fluorescence intensity and expression patterns were observed for the functional protein marker Na^+^/K^+^-ATPase and surface glycoproteins NCAM and GPC4. Similar observations have been reported in other studies where the authors concluded that ROCK-inhibitors did not induce proliferation or alter the apoptosis of human CECs in culture but modulated the cell adhesion properties, cell morphology, and cell junctions by regulating the dynamic rearrangements of the actin cytoskeleton [34,35].

It is possible, however, that the growth factors present in the serum and routinely added to these explant cell cultures already promote cell growth to the point that the effect of a ROCK-inhibitor cannot be detected. Another limitation of our analysis is the small number of outer graft rims used to test whether the ROCK-inhibitor influences the migratory ability of corneal endothelial cells *in vitro* and larger studies are needed to obtain more conclusive results under which conditions ROCK-inhibitor is most effective in stimulating cell migration. A technical limitation was the challenging observation of the late-onset cells, that were located close to the borders of the well-plate, due to an uneven illumination caused by liquid meniscus. In addition, since experiments were conducted with the trabecular meshwork still attached to the corneal rings, the origin of the late-onset cells cannot be unequivocally established with the current set-up; and a follow-up study with paired rings with and without trabecular meshwork attached followed by quantitative polymerase chain reaction (q-PCR) analysis may provide more conclusive information on the origin of this cell type.

## Conclusion

In conclusion, we present the findings of selectively studying CEC migration from the peripheral cornea by using outer graft rims embedded into a thermo-reversible polymer matrix. In this study, we observed the migration of two morphologically distinct cell populations. The first type was triggered by a broken physical barrier, while the second, late-onset type appeared less differentiated but showed a higher proliferative capacity. It is possible that the activation of the later cell population that displays characteristics of the TZ cells is governed by stimuli other than the loss of contact inhibition and the influence of a ROCK-inhibitor. Understanding the cell migration mechanism from phenotypically distinct regions of the endothelium may assist in optimizing regenerative therapies for endothelial diseases and whether other means of pharmaceutical modulation could further improve the outcomes.

## Supporting information

S1 Fig Light microscopy imaging overview of all donor grafts. Light microscopy examination of the donor corneas performed by the eye bank before graft preparation. For the proof-of-concept cell migration study, grafts of corneas #1 to #7 (Table 1) were used. Grafts used for the paired outer graft rims cell migration study were prepared from corneas # 8 to #21. The corneal endothelium from #14, #15, and #21 showed initially poor visualization, but images (inserts, x200) taken after graft preparation show the presence of an endothelial monolayer.
(PDF)

S2 Fig Control graft—outer rim pair showing different cell migration behavior. (A) Light microscopy overview collage (x50) of a control graft cultured for 19 days in a hydrogel matrix showing the formation of a continuous monolayer. Higher magnification images from the areas marked by * and ** in the overview image illustrate a contact inhibited cell monolayer formed around the entire circular graft edge up to the cell migration edge. (B) Composite photos (x50) stitched together to create an image panorama of the outer rim stained with Calcein-AM after 47 days of gel culture. Note the imprints (market by white arrows) left by the surgical blade on the well surface after cutting open the outer rim. Initial endothelial cell density of the donor cornea was 2200 cells/mm^2^. Scale bars in (A) and (B): 1000 μm, and in higher magnification images:100 μm.
(PDF)

S3 Fig Fluorescence imaging overviews. Overviews (collage of x50) are shown for two unpaired outer rims stained with Calcein-AM and cultured (A) in the presence or (B) absence of ROCK-inhibitor. Both outer rims showed cells migrating from areas with low cell viability. Higher magnification images labeled with (A1) to (A4) in the graft overview from (A) represent double staining Calcein-AM(green)/PI(red) showing viable cells (green only), cells in late apoptosis with a damaged membrane (low green signal + strong red signal), necrotic cells (strong red signal only). A significant occurrence of cell migration was initiated from areas with a low density of viable cells. Scale bars in (A) and (B): 1000 μm, and in higher magnification images (A1) to (A4): 100 μm.
(PDF)
