# Supplementary material for: Early and late-onset cell migration from peripheral corneal endothelium
Source: PLoS One. 2023 May 10;18(5):e0285609. doi: 10.1371/journal.pone.0285609 (PMC10171599; doi:10.1371/journal.pone.0285609)
Supplement: S1 Fig — Light microscopy examination of the donor corneas performed by the eye bank before graft preparation. For the proof-of-concept cell migration study, grafts of corneas #1 to #7 (Table 1) were used. Grafts used for the paired outer graft rims cell migration study were prepared from corneas # 8 to #21. The corneal endothelium from #14, #15, and #21 showed initially poor visualization, but images (inserts, x200) taken after graft preparation show the presence of an endothelial monolayer. (PDF) [file pone.0285609.s001.pdf]

Supporting Information

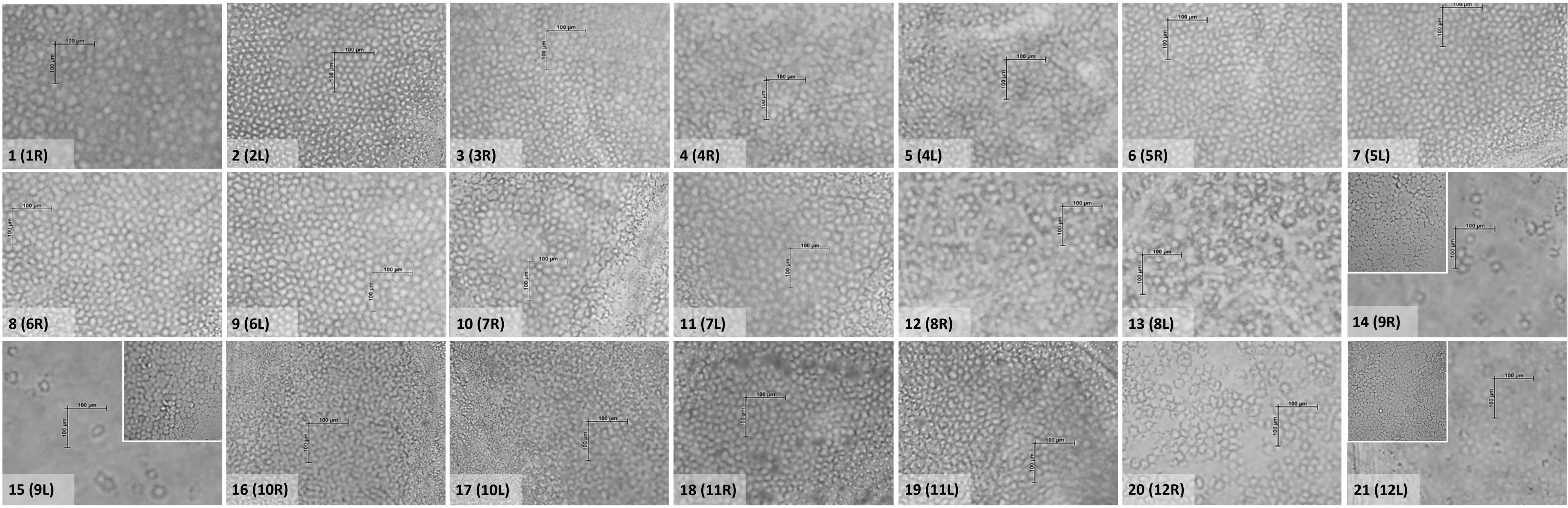

S1 Fig. Light microscopy imaging overview of all donor grafts. Light microscopy examination of the donor corneas performed by the eye bank before graft preparation. For the proof-of-concept cell migration study, grafts of corneas #1 to #7 (Table 1) were used. Grafts used for the paired outer graft rims cell migration study were prepared from corneas # 8 to #21. The corneal endothelium from #14, #15, and #21 showed initially poor visualization, but images (inserts, x200) taken after graft preparation show the presence of an endothelial monolayer.
