# Supplementary material for: Early and late-onset cell migration from peripheral corneal endothelium
Source: PLoS One. 2023 May 10;18(5):e0285609. doi: 10.1371/journal.pone.0285609 (PMC10171599; doi:10.1371/journal.pone.0285609)
Supplement: S2 Fig — (A) Light microscopy overview collage (x50) of a control graft cultured for 19 days in a hydrogel matrix showing the formation of a continuous monolayer. Higher magnification images from the areas marked by * and ** in the overview image illustrate a contact inhibited cell monolayer formed around the entire circular graft edge up to the cell migration edge. (B) Composite photos (x50) stitched together to create an image panorama of the outer rim stained with Calcein-AM after 47 days of gel culture. Note the imprints (market by white arrows) left by the surgical blade on the well surface after cutting open the outer rim. Initial endothelial cell density of the donor cornea was 2200 cells/mm2. Scale bars in (A) and (B): 1000 μm, and in higher magnification images:100 μm. (PDF) [file pone.0285609.s002.pdf]

Supporting Information

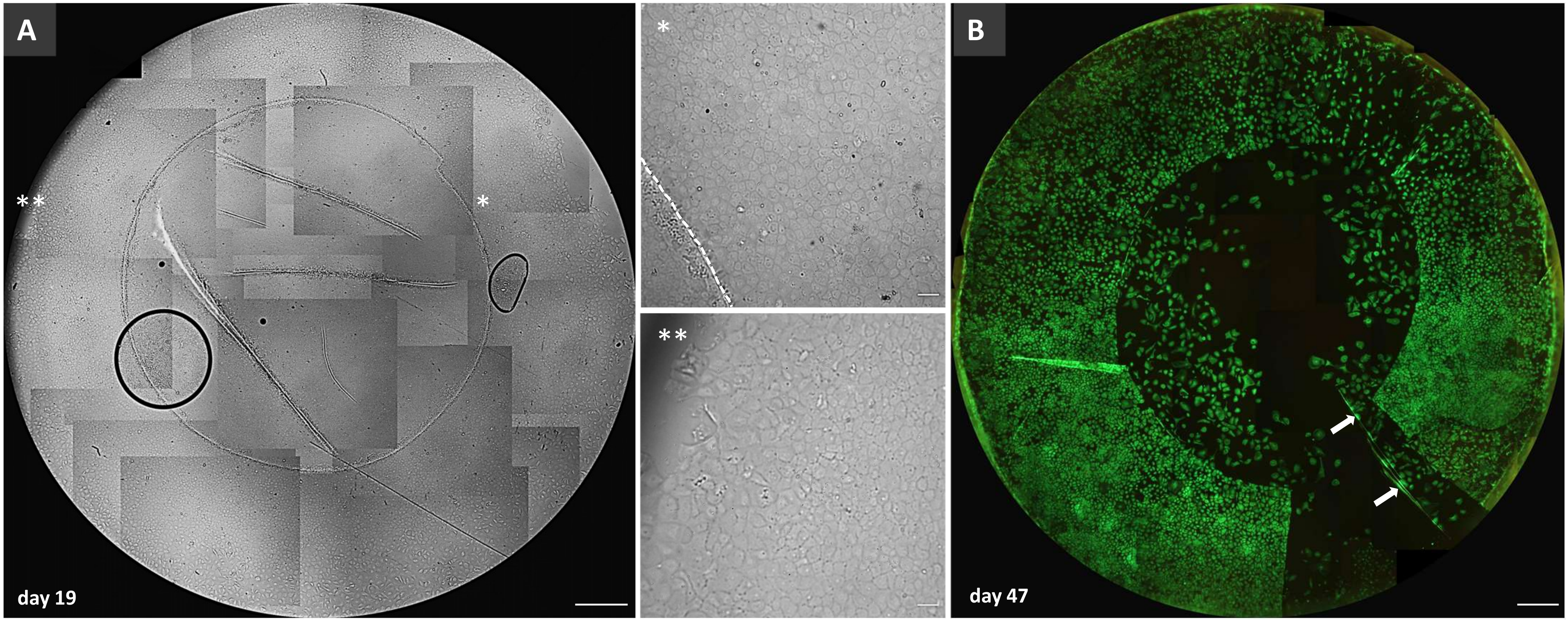

S2 Fig. Control graft - outer rim pair showing different cell migration behavior. (A) Light microscopy overview collage (x50) of a control graft cultured for 19 days in a hydrogel matrix showing the formation of a continuous monolayer. Higher magnification images from the areas marked by * and ** in the overview image illustrate a contact inhibited cell monolayer formed around the entire circular graft edge up to the cell migration edge. (B) Composite photos (x50) stitched together to create an image panorama of the outer rim stained with Calcein-AM after 47 days of gel culture. Note the imprints (market by white arrows) left by the surgical blade on the well surface after cutting open the outer rim. Initial endothelial cell density of the donor cornea was 2200 cells/mm^2. Scale bars in (A) and (B): 1000 µm, and in higher magnification images:100 µm.
