# Supplementary material for: Early and late-onset cell migration from peripheral corneal endothelium
Source: PLoS One. 2023 May 10;18(5):e0285609. doi: 10.1371/journal.pone.0285609 (PMC10171599; doi:10.1371/journal.pone.0285609)
Supplement: S3 Fig — Overviews (collage of x50) are shown for two unpaired outer rims stained with Calcein-AM and cultured (A) in the presence or (B) absence of ROCK-inhibitor. Both outer rims showed cells migrating from areas with low cell viability. Higher magnification images labeled with (A1) to (A4) in the graft overview from (A) represent double staining Calcein-AM(green)/PI(red) showing viable cells (green only), cells in late apoptosis with a damaged membrane (low green signal + strong red signal), necrotic cells (strong red signal only). A significant occurrence of cell migration was initiated from areas with a low density of viable cells. Scale bars in (A) and (B): 1000 μm, and in higher magnification images (A1) to (A4): 100 μm. (PDF) [file pone.0285609.s003.pdf]

Supporting Information

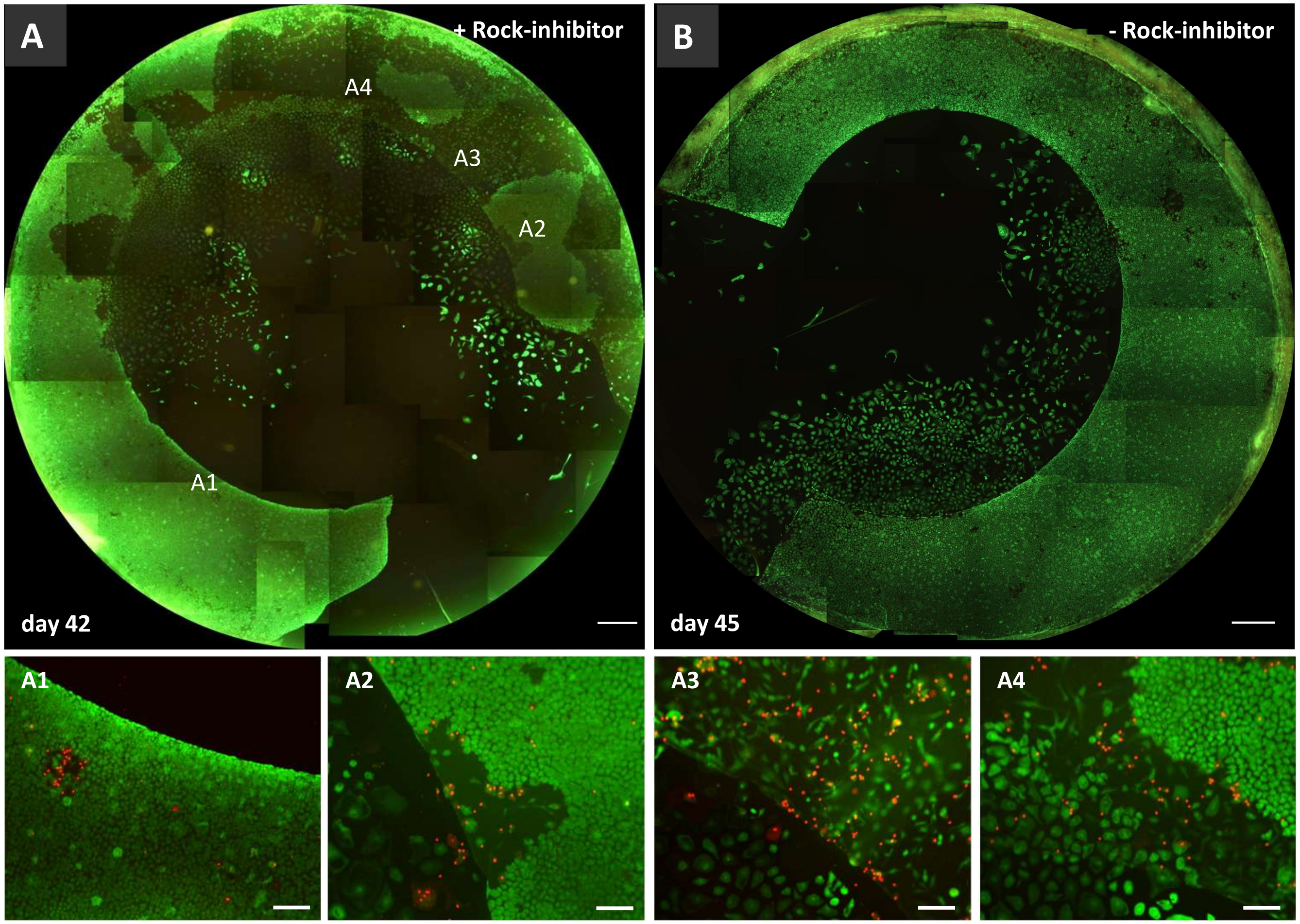

S3 Fig. Fluorescence imaging overviews. Overviews (collage of x50) are shown for two unpaired outer rims stained with Calcein-AM and cultured (A) in the presence or (B) absence of ROCK-inhibitor. Both outer rims showed cells migrating from areas with low cell viability. Higher magnification images labeled with (A1) to (A4) in the graft overview from (A) represent double staining Calcein-AM(green)/PI(red) showing viable cells (green only), cells in late apoptosis with a damaged membrane (low green signal + strong red signal), necrotic cells (strong red signal only). A significant occurrence of cell migration was initiated from areas with a low density of viable cells. Scale bar (A) and (B): 1000 µm. Scale bar (A1–A4): 100 µm.
